# MicroRNA 483-3p targets Pard3 to potentiate TGF-β1-induced cell migration, invasion, and epithelial–mesenchymal transition in anaplastic thyroid cancer cells

**DOI:** 10.1038/s41388-018-0447-1

**Published:** 2018-08-31

**Authors:** Xiaoping Zhang, Lin Liu, Xianzhao Deng, Dan Li, Haidong Cai, Yushui Ma, Chengyou Jia, Bo Wu, Youben Fan, Zhongwei Lv

**Affiliations:** 1 Department of Nuclear Medicine, Shanghai Tenth People’s Hospital, Tongji University, Shanghai, 200072 China; 2 Shanghai Center of Thyroid Diseases, Shanghai, 200072 China; 3 Center of Thyroid, Department of General Surgery, Shanghai Jiao Tong University Affiliated Sixth People’s Hospital, Shanghai, 200233 China

**Keywords:** Oncogenes, Cell migration

## Abstract

Anaplastic thyroid cancer (ATC) is associated with poor prognosis and is often untreatable. MicroRNA 483-3p (miR-483) and partitioning-defective 3 (Pard3), a member of the Pard family, have functions and regulatory mechanisms in ATC. The abnormal regulation of miR-483 may play an important role in tumorigenesis, and Par3 is known to regulate cell polarity, cell migration, and cell division. Tumor proliferation promoted by the regulation of miRNA expression can be regulated in thyroid cancer by upregulating transforming growth factor-β1 (TGF-β1), which is thought to interact with Pard3. When compared with adjacent non-tumor tissues, we found that miR-483 was upregulated and Pard3 was downregulated in 80 thyroid tumor samples. Disease-free survival was decreased when expression of miR-483 was upregulated and Pard3 expression was downregulated. Cell growth, migration, and invasion were induced by overexpression of miR-483. However, knockdown of miR-483 resulted in a loss of cell invasion and viability, both in vitro and in vivo. The expression of Pard3 was increased by the inhibition of miR-483, but TGF-β1-induced cell migration and invasion were decreased by miR-483 inhibition. A dual-luciferase reporter assay determined that Pard3 expression was downregulated when targeted with miR-483. The epithelial–mesenchymal transition (EMT), as well as Tiam1-Rac signaling, was induced by TGF-β1, which was decreased by the overexpression of Pard3. Pard3 decreased the inhibition of EMT and Tiam-Rac1 signaling, which resulted from transfection of ATC cells with miR-483. Overall, the results showed that downregulation of Pard3 resulted in increased cell invasion and EMT in ATC, which was promoted by treatment with miR-483. These findings suggest novel therapeutic targets and treatment strategies for this disease.

## Introduction

During the last few decades, the incidence of thyroid cancer has increased in many countries [1, 2]. The four classifications of thyroid tumors include well-differentiated tumors such as papillary and follicular carcinomas, poorly differentiated tumors, and undifferentiated tumors such as anaplastic carcinomas. The majority of thyroid tumors are differentiated and these tumors respond well to therapy. Anaplastic thyroid cancer (ATC), however, is associated with poor prognosis and many times is not treatable [3]. Understanding the underlying molecular mechanisms of undifferentiated thyroid cancer will help to develop novel targeted therapies and improved patient survival.

MicroRNAs (miRNAs) are non-coding RNAs, which are many times abnormally regulated in human tumors. Many miRNAs have been studied as potential biomarkers and therapeutic targets of different cancers [4–6]. MiR-483 is overexpressed in many types of cancers [7], including esophageal squamous cell carcinoma [6], hepatocellular carcinoma [8], pancreatic cancer [9]; miR-483 promotes the epithelial–mesenchymal transition (EMT), invasion, and metastasis in lung adenocarcinoma [10]. The downregulation of tumor suppressor genes in these cancers results in oncogenesis that is promoted by miR-483. Nonetheless, little is known about the role played by miR-483 in ATCs, as well as its effects on target genes.

Smads interact with transforming growth factor-β1 (TGF-β1), which is a member of a family of cytokines involved in cell growth, differentiation, and apoptosis. The interaction then results in translocation of Smads to the nucleus, where they can alter gene expression. TGF-β1 and Smad activity is upregulated in thyroid carcinomas [11, 12]. Furthermore, TGF-β1 promotes EMT, and increases metastasis and tumor growth by regulating miRNAs in different cancers [11–14].

Cell polarity, migration, and division are regulated by the partitioning-defective (Pard) protein family [15]. Disruption of cell polarity in many types of cancers has been thought to be affected by the dysfunction of the Pard complex. Pard3 is thought to interact with TGF-β, resulting in changes in the Pard complex, resulting in a complex that induces transformation instead of regulation of normal polarity [16]. The Rac1-specific guanine nucleotide exchange factor (GEF), Tiam1, interacts with Pard3 of the Pard family by its protein interaction domain, resulting in a decrease in Tiam1-mediated Rac1 activation [17]. Increased Tiam1-mediated Rac activation results from a decrease in Pard3 in tumors, followed by disruption of actin dynamics and a decrease in cell–cell junctions [18–22]. These Tiam1/Rac1/MAPK pathway mechanisms promote tumorigenesis and metastasis in breast cancer [19, 21], lung squamous cell carcinoma [18], pancreatic cancer [20], lung adenocarcinoma [22], and prostate cancer [23].

The role of miR-483 in ATCs was characterized in the present study. We showed that TGF-β1-induced EMT in ATC cells was mediated by miR-483, involving direct binding to Pard3, to affect Tiam/Rac1 signaling. Overall, our results provided a better understanding of the molecular mechanisms involved in ATC, which will assist in the development of novel therapeutic agents for the treatment of this disorder.

## Results

### MiR-483 and Pard3 expression in thyroid cancer tissues and ATC cell lines

**Fig. 1:**
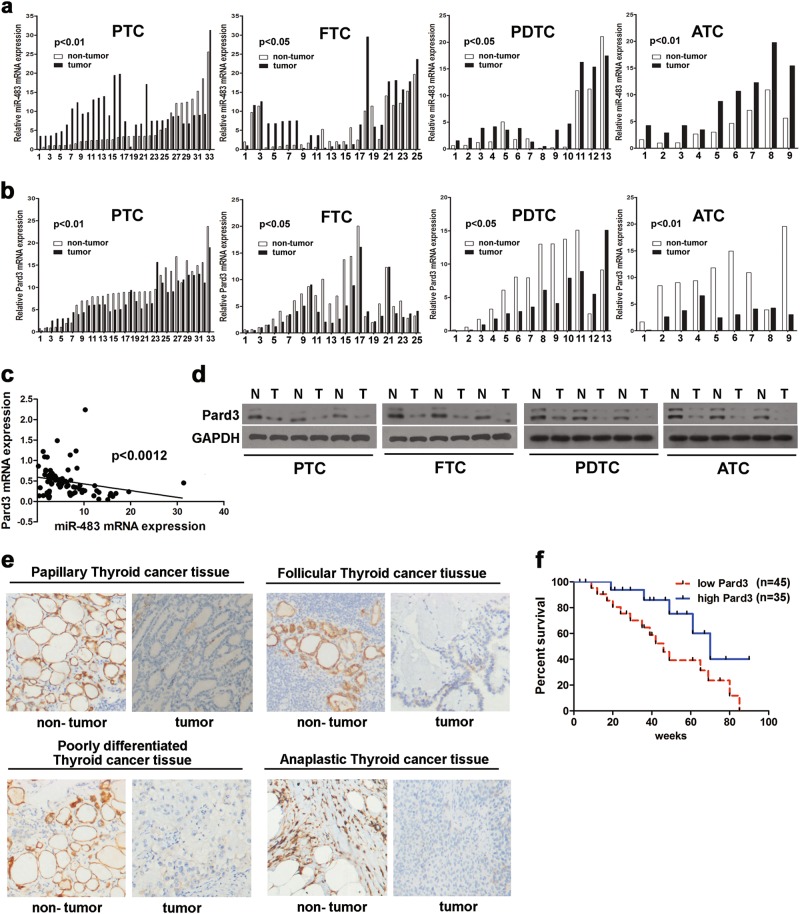
MiR-483 and Pard3 expression in tumor tissues. **a**, **b** MiR-483 and Pard3 expression in 80 paired thyroid cancer tumor and adjacent non-tumor tissues analyzed by qRT-PCR (*n* = 80, *p < 0.05, **p < 0.01, Student’s *t* test). **c** The relationship of Pard3 expression and miR-483 expression was detected by Spearman’s correlation analyses (*r* = −0.3548, **p *<* 0.0012). **d** Pard3 protein expression was analyzed by western blots in four thyroid cancer tissues (PTC, FTC, PDTC, and ATC). **e** Pard3 expression was analyzed by immunohistochemistry in four thyroid tissues, respectively. **f** The Kaplan–Meier method was used to analyze the relationship between Pard3 expression and survival of thyroid cancer patients (*p *<* 0.0429, log-rank test). T tumor, N non-tumor

Using histological analyses of tissue from 80 patients, four types of thyroid cancer were found (papillary (*n* = 33) and follicular (*n* = 25) carcinomas, poorly differentiated tumors (*n* = 13), and anaplastic carcinomas (*n* = 9)). The clinicopathological characteristics of Pard3 “low” or “high” expression are detailed in Table 1. Figure 1a shows that when compared with adjacent normal thyroid tissues, the expression of miR-483 was higher in the four types of cancers, including papillary thyroid cancer (PTC), follicular thyroid cancer (FTC), poorly differentiated thyroid cancer (PDTC), and ATC tissues. However, Fig. 1b shows that when compared with PTC, FTC, PDTC, and ATC tissues, adjacent normal thyroid tissue contained higher expression of Pard3. Figure 1c, d show that miR-483 and Pard3 expressions were negatively correlated, and there were decreased Pard3 levels in all types of thyroid cancers. Pard3 expression was detected in the four different types of thyroid cancer tissue by immunohistochemistry (IHC) (Fig. 1e). Non-tumor sections showed positive Pard3 staining, while tumor tissues showed a negative expression. In thyroid cancer patients, a decreased disease-free survival was correlated with a reduction in Pard3 expression, when using Kapan–Meier analyses (Fig. 1f). The areas under the receiver operating characteristic curve using miR-483 were 0.740 with a 95% confidence interval (CI) of 0.631–0.850 (*p* = 0.002). In thyroid cancer patients, there was decreased disease-free survival, which was associated with increased miR-483 expression (Supplementary Figure 1a and b).

**Table 1:** Pard3 staining and clinicopathological characteristics of 80 thyorid cancer patients

Parameters	Pard3		Total	*P* value
	Low (%)	High (%)		
*Age (years old)*				0.167
<50	20 (25)	21 (26.25)	41	
≥50	25 (31.25)	14 (17.5)	39	
*Gender*				0.149
Male	24 (30)	13 (16.25)	37	
Female	21 (26.25)	22 (27.5)	43	
*Histopathology*				0.851
Papillary	20 (25)	13 (16.25)	33	
Follicular	14 (17.5)	11 (13.75)	25	
Poorly differentiated	7 (8.75)	6 (7.5)	13	
Anaplastic	4 (5)	5 (6.25)	9	
*TNM stage*				0.040*
I–II	13 (16.25)	18 (22.5)	31	
III–IV	32 (40)	17 (21.25)	49	

*p *<* 0.05, *χ*^2^ test

### The in vitro promotion of proliferation, migration, and invasion of ATC cells by miR-483

**Fig. 2:**
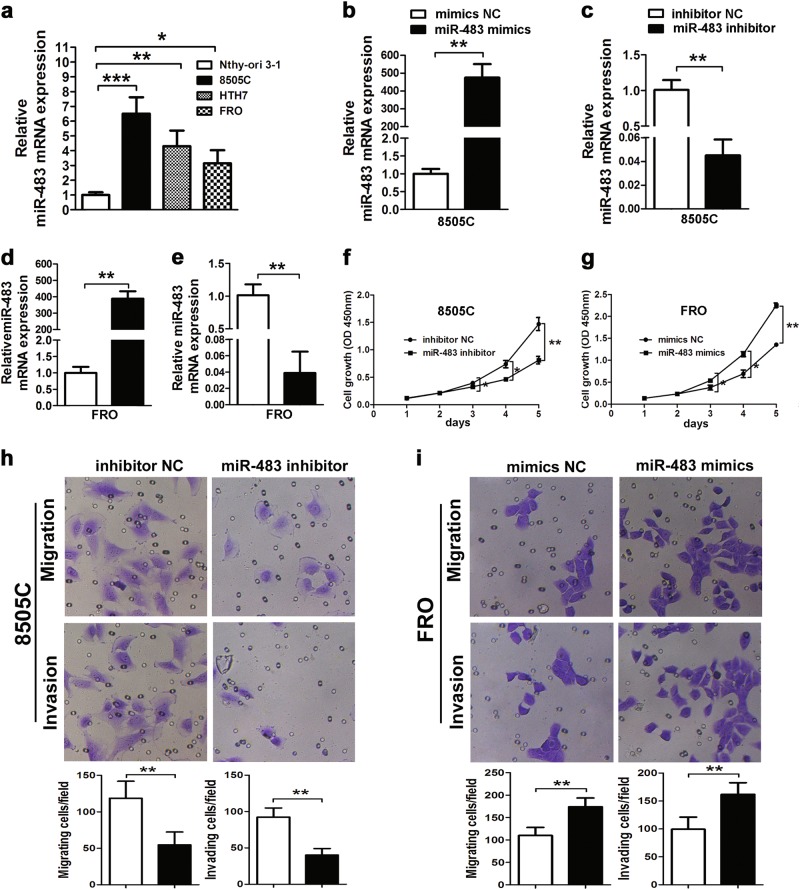
MiR-483 promotes ATC cell proliferation, migration, and invasion in vitro. **a** The qRT-PCR analyses of miR-483 expression in ATC cell lines 8505C, HTH7, FRO, and the normal thyroid cell line, Nthy-ori 3-1 (*p *<* 0.05, **p *<* 0.01, ***p *<* 0.001, one-way analysis of variance). **b**–**e** The 8505C and FRO cells were transfected with miR-483 mimics/mimics NC or miR-483 inhibitors/inhibitor NC. MiR-483 was detected by qRT-PCR (**p *<* 0.01, Student’s *t* test). **f**, **g** The 8505C cells were transfected with miR-483 inhibitors and FRO cells were transfected with miR-483 mimics. Cell growth was determined by the CCK-8 assay (*p *<* 0.05, **p *<* 0.01, one-way analysis of variance). **h**, **i** Cell migration and invasion were assessed by Transwell^®^ assays (**p *<* 0.01, Student’s *t* test). *N* = 3 independent experiments with triplicate biological replicates for each line

The expression of miR-483 was quantitated in normal thyroid cells, as well as in three ATC cell lines. Figure 2a shows that when compared with normal thyroid cells, the expression of miR-483 was increased in ATC cells. We used commercially obtained miR-483 mimics for miR-483 overexpression or miR-483 inhibitors for miR-483 knockdown to significantly upregulate or downregulate miR-483 in 8505C and FRO cells (Fig. 2b–e). FRO cells were transfected with miR-483 mimics, and 8505C cells were transfected with miR-483 inhibitors, to determine the effects of miR-483 on ATC cells. Figure 2f, g shows that the proliferation of ATC cells was significantly reduced when miR-483 was inhibited, while ATC cell proliferation was significantly increased by miR-483 mimics. ATC cell migration and invasion abilities were investigated using transwell assays. When compared with control cells, the migration and invasion of 8505C cells were significantly reduced when miR-483 was inhibited (Fig. 2h). However, Fig. 2i shows that when compared with controls, cell migration and invasion of FRO cells were significantly increased when treated with miR-483 mimics. In addition, Supplementary Figure 1c and d show that the growth, migration, and invasion of normal thyroid cells were increased when treated with miR-483.

### The in vivo promotion of tumor growth by miR-483

**Fig. 3:**
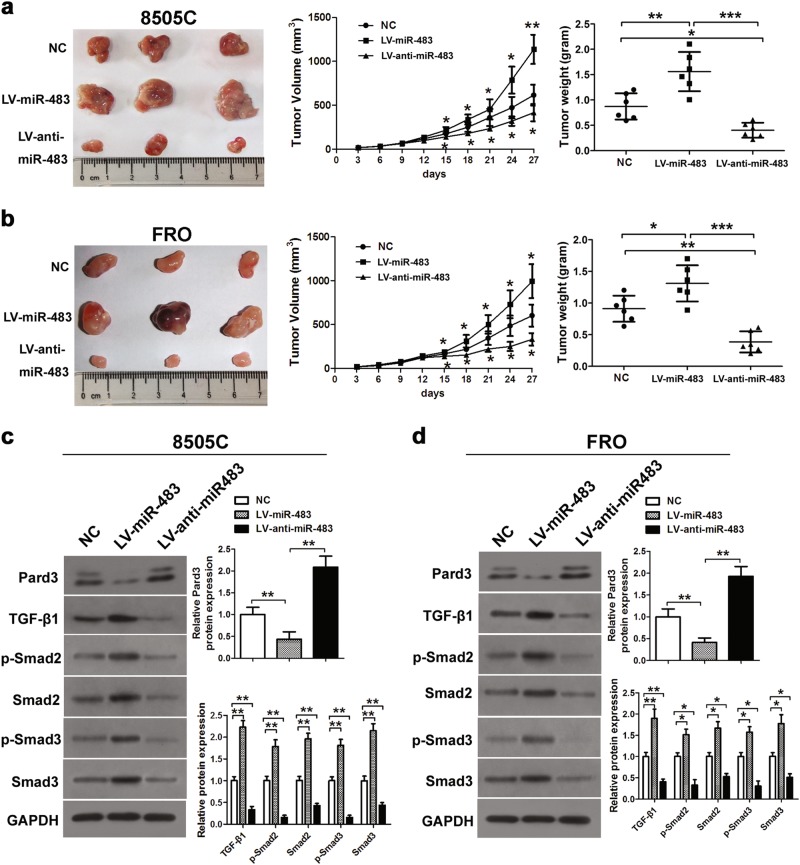
MiR-483 promotes tumor growth in vivo. Photographs of tumors derived from mice 27 days after implantation of 8505C (**a**) or FRO (**b**) cells transfected with LV-miR-483 or LV-anti-miR-483. The average tumor weight was measured and growth curves were generated (*n* = 6, *p *<* 0.05, **p *<* 0.01, ***p *<* 0.001, one-way analysis of variance). **c**, **d** Western blotting was used to analyze Pard3 expression and TGF-β1, p-Smad2, Smad2, p-Smad3, and Smad3 in LV-miR-483 or LV-anti-miR-483 8505C cell tumors or FRO cell tumors, respectively (*p *<* 0.05, **p *<* 0.01, ***p *<* 0.001, one-way analysis of variance)

Using retroviral infection, LV-miR-483 or LV-anti-miR-483 constructs were introduced into FRO and 8505C cells, in order to determine the possible effect of miR-483 on ATC cells. We then injected these cells into mice to induce tumor formation. Compared with control cells, the excised tumors were larger from mice injected with the miR-483-expressing cells. However, in animals injected with the LV-anti-miR-483-infected cells, the tumors were significantly smaller. Quantification of tumor size and tumor weight (Fig. 3a, b) confirmed these observations, suggesting that miR-483 knockdown inhibited tumor growth. We also used 8505C and FRO cells to assess Pard3 protein levels and TGF-β1 signaling expression (Fig. 3c, d). TGF-β1 was significantly reduced in 8505C cells after miR-483 inhibition. The levels of TGF-β1, p-Smad2, Smad2, p-Smad3, and Smad3 were significantly increased during the overexpression of miR-483, when both types of cells were treated. Overall, these results showed that the signaling of TGF-β1 was increased when the levels of Pard3 were reduced.

### TGF-β1 increased miR-483 expression and inhibited Pard3 expression

**Fig. 4:**
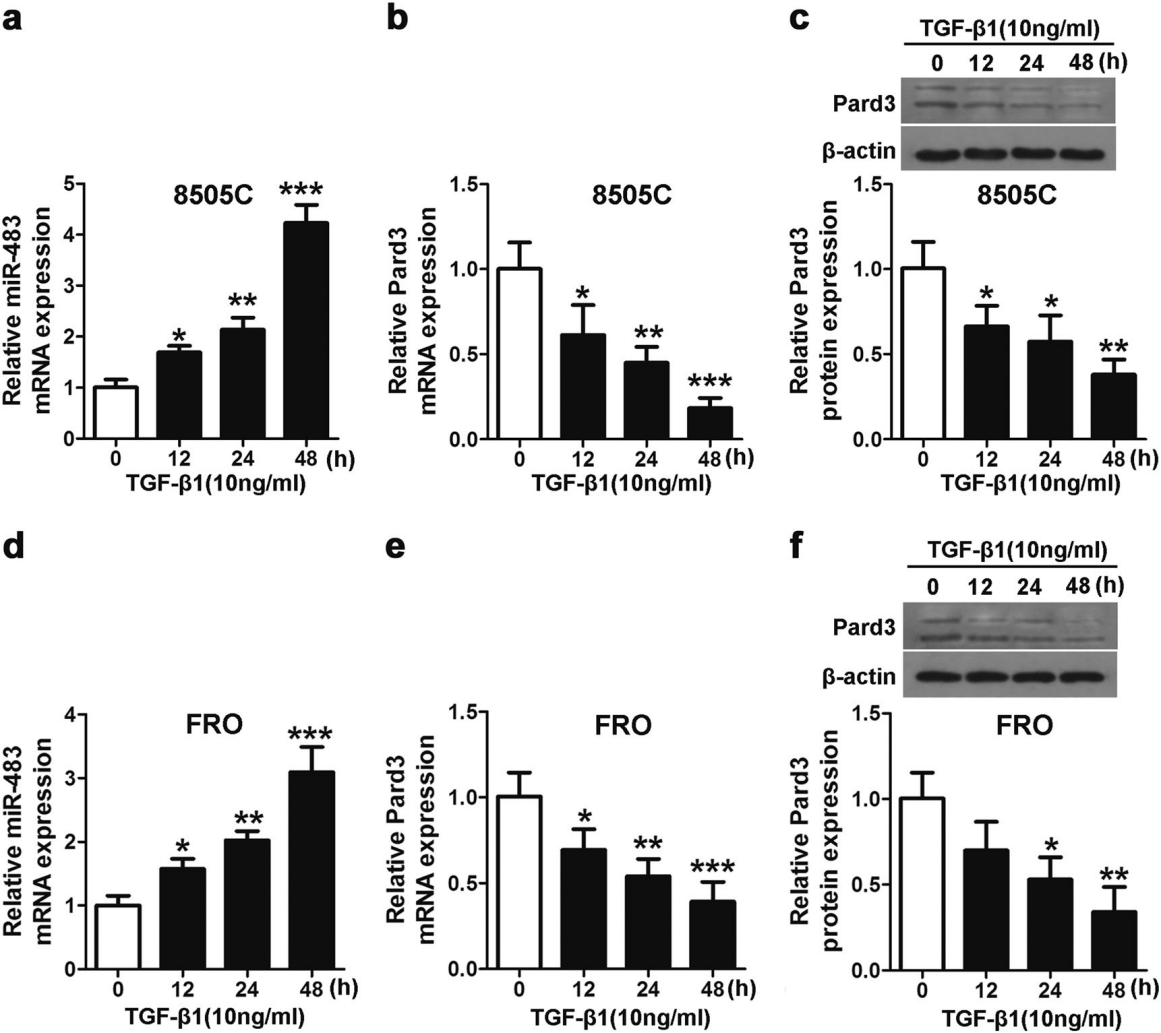
TGF-β1 increases miR-483 expression and decreases Pard3 expression. **a**, **d** qRT-PCR analyses of miR-483 expression after TGF-β1 treatment (10 ng/mL) in 8505C or FRO cells. Pard3 mRNA (**b**, **c**) and protein expression (**e**, **f**) following TGF-β1 treatment (10 ng/mL) (*p < 0.05, **p < 0.01, ***p < 0.001, one-way analysis of variance). *N* = 3 independent experiments with triplicate biological replicates for each line

The effects of TGF-β1 treatment on the expressions of miR-483 and Pard3 were then determined. Figure 4a–f shows that when 8505C and FRO cells were treated with 10 ng/mL TGF-β1, there was a significant increase in miR-483 expression, as well as a significant reduction of Pard3 messenger RNA (mRNA) and protein, both of which occurred in a time-dependent manner.

### Downregulation of miR-483 inhibited TGF-β1-induced cell migration and invasion of ATC cells in vitro

**Fig. 5:**
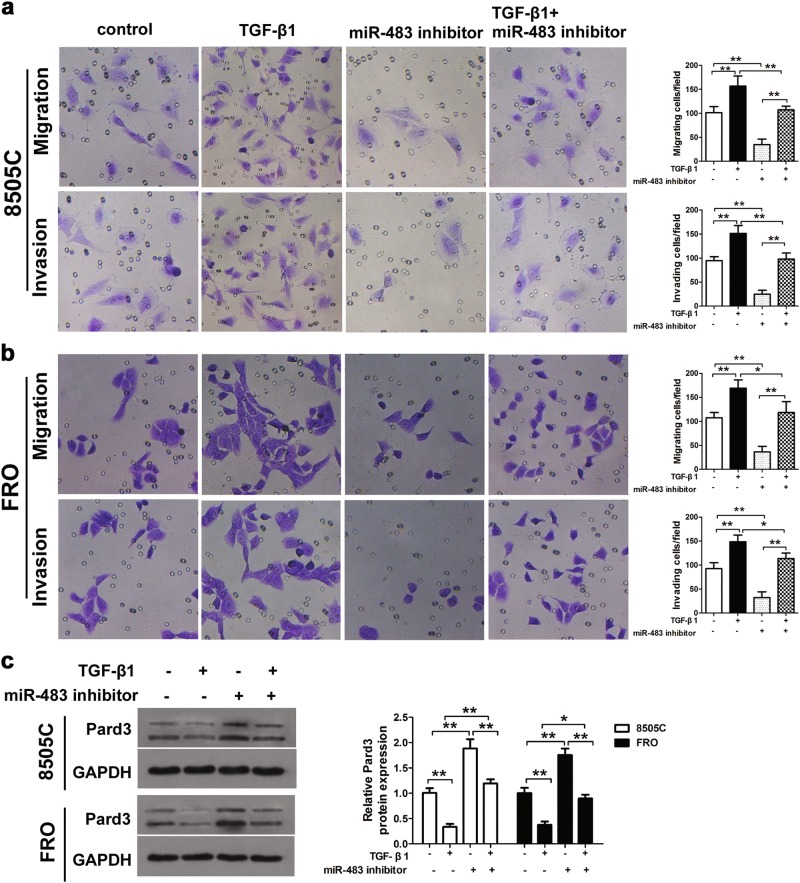
MiR-483 inhibition prevents TGF-β1-induced ATC cell migration and invasion. **a**, **b** The 8505C or FRO cells were transfected with miR-483 inhibitor or miR-483 inhibitor NC, and subsequently treated with TGF-β1 (10 ng/mL) for 48 h. Untransfected cells with or without TGF-β1 treatment were also included. ATC cell migration and invasion were measured by Transwell^®^ assays. **c** Western blots were used to detect Pard3 protein expression. GAPDH was used as a loading control (*p < 0.05, **p *<* 0.01, one-way analysis of variance). *n* = 3 independent experiments with triplicate biological replicates for each line

The 8505C and FRO cells were transfected with miR-483 inhibitors, then treated with 10 ng/mL TGF-β1, to determine the effects of miR-483 and TGF-β1 on ATC cells. Transwell assays were then used to determine the cell migration and invasion of thyroid cancer cells. Figure 5a, b show that there was increased migration and invasion, when compared with control cells, when 8505C and FRO cells were treated with TGF-β1. Furthermore, when miR-483 was downregulated, these effects were decreased. Pard3 protein expression was downregulated by TGF-β1 treatment and transfection with a miR-483 inhibitor attenuated these effects (Fig. 5c).

### MiR-483 targets Pard3 3′-UTR

**Fig. 6:**
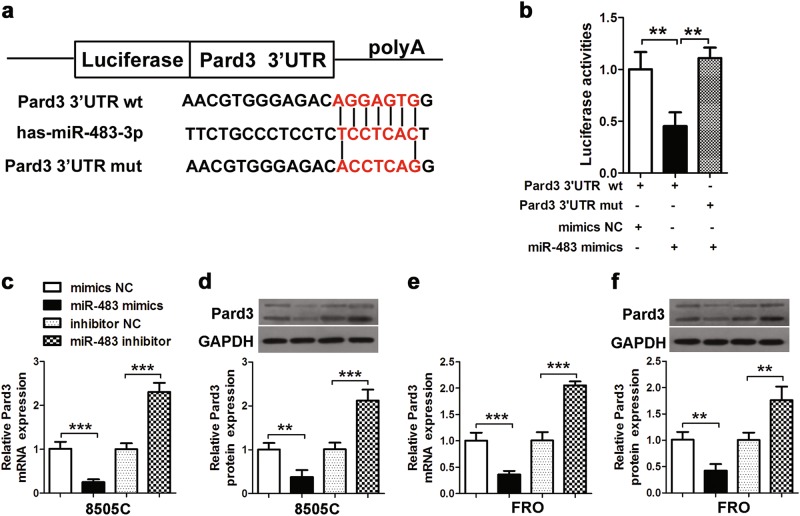
MiR-483 targets Pard3. **a** Putative miR-483 binding sites in the Pard3 3′-UTR. **b** A luciferase reporter plasmid containing wild-type or mutant Pard3 was co-transfected into HEK293T cells with miR-483 mimics or mimics NC. Luciferase activity was determined at 48 h after transfection using the dual-luciferase assay and was normalized to Renilla luciferase activity. **c–f** qRT-PCR and western blot assessment of Pard3 expression in 8505C or FRO cells transfected with miR-483 mimics, mimics NC, miR-483 inhibitor, and inhibitor NC. GAPDH was used as a loading control. **p < 0.01, ***p < 0.001, one-way analysis of variance. *N* = 3 independent experiments with triplicate biological replicates for each line

Putative binding sites on the 3′-untranslated region (3′-UTR) of Pard3 (Fig. 6a) by miR-483 were identified to determine the possible relationship between miR-483 and Pard3 (Fig. 6a). This identification involved muted binding sites on luciferase reporter constructs. Using miR-483 mimics or controls (miR-NC), 293T cells were transfected with mutant (mut) and wild-type (wt) luciferase reporter constructs. Figure 6b shows that in cells transfected with wt constructs, miR-483 mimics significantly inhibited luciferase activity; however, luciferase activity was unaffected in cells that were transfected with the mut constructs. These results showed that Pard3 was directly targeted by miR-483. When compared with controls, Pard3 expression was significantly downregulated in 8505C and FRO cells when treated with miR-483 mimics, while Pard3 expression was significantly upregulated when treated with miR-483 inhibitors (Fig. 6c–f). Overall, the results showed that Pard3 and miR-483 directly interact, resulting in the downregulation of Pard3 in ATC cells.

### Pard3 mediates TGF-β1-induced EMT and activation of Tiam1-Rac1 signaling in ATC cells in vitro

**Fig. 7:**
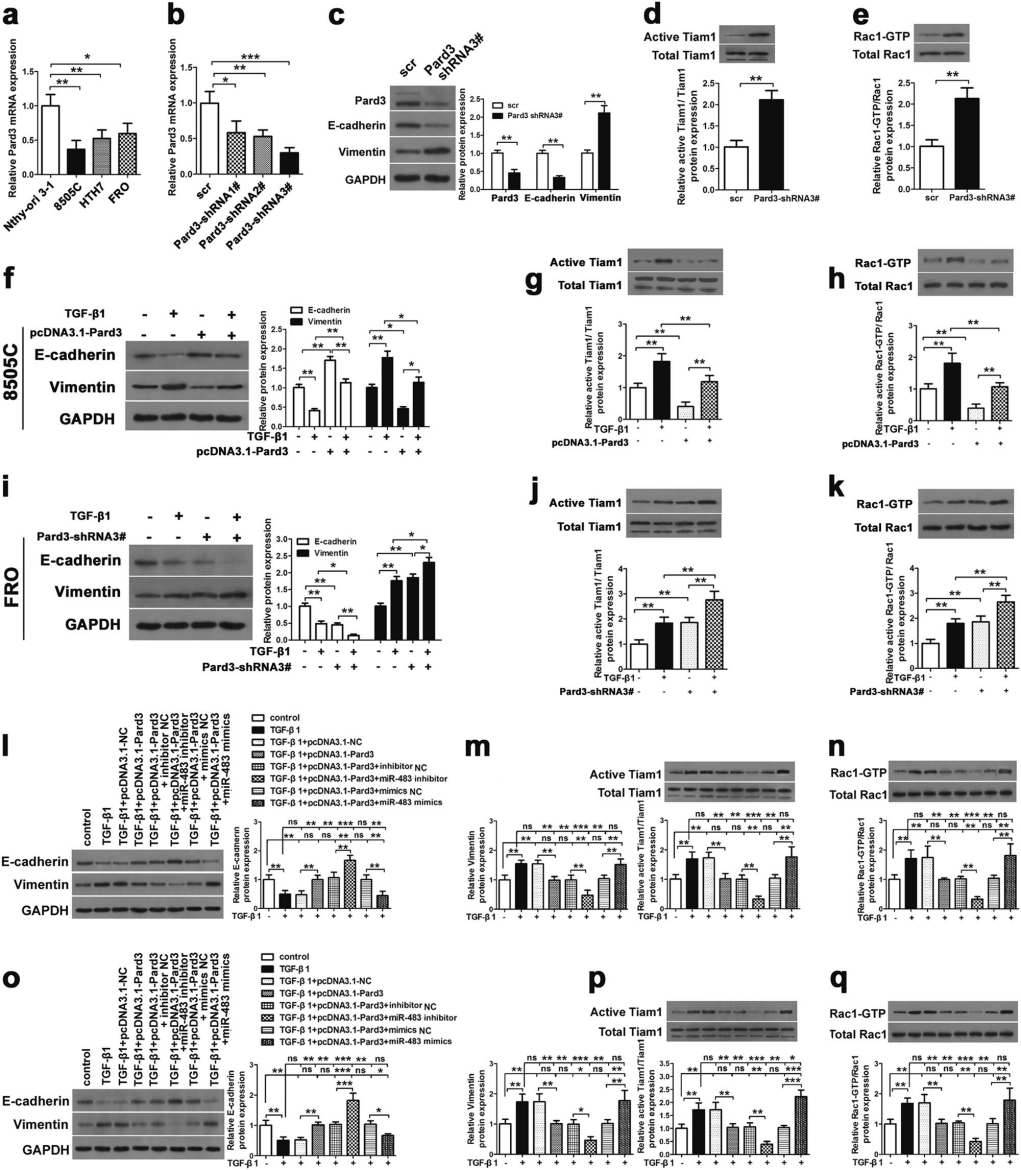
MiR-483 promotes TGF-β1-induced EMT and Tiam1/Rac signaling by downregulating Pard3. **a** Pard3 mRNA expression in the ATC cell lines, 8505C, HTH7, and FRO, and normal thy-ori 3-1 cells (*p *<* 0.05, **p *<* 0.01, one-way analysis of variance). **b** Knockdown of Pard3 (Pard3-shRNA1–3) in FRO cells and qRT-PCR assessment of Pard3 expression (*p *<* 0.05, **p *<* 0.01, ***p *<* 0.001, one-way analysis of variance). **c**–**e** FRO cells were stably transfected with Pard3-shRNA3 or scr. E-cadherin, vimentin, Tiam1, and Rac1 expression were detected by western blotting. GAPDH was used as a loading control (**p *<* 0.01, Student’s *t* test). The 8505C cells were stably transfected with pcDNA3.1-Pard3 and subsequently treated with TGF-β1 (10 ng/mL) for 48 h. Untransfected cells with or without TGF-β1 treatment were also included. **f**–**h** E-cadherin, vimentin, Tiam1, and Rac1 expression were detected by western blotting. GAPDH was used as a loading control (*p *<* 0.05, **p *<* 0.01, one-way analysis of variance). FRO cells were transfected with Pard3-shRNA3, and subsequently treated with TGF-β1 (10 ng/mL) for 48 h. Untransfected cells with or without TGF-β1 treatment were also included. **i**–**k** E-cadherin, vimentin, Tiam1, and Rac1 expression were detected by western blotting. GAPDH was used as a loading control (*p < 0.05, **p < 0.01, one-way analysis of variance). ATC cells stably transfected with pcDNA3.1-NC or pcDNA3.1-Pard3 were transfected with miR-483 inhibitor/miR-483 inhibitor NC, or miR-483 mimics/miR-483 mimics NC, and subsequently treated with TGF-β1 (10 ng/mL) for 48 h. Untransfected cells with or without TGF-β1 treatment were also included. E-cadherin, Tiam1, and Rac1 expression were detected by western blotting in both 8505C cells (**l**–**n**) and FRO cells (**o**–**q**). GAPDH was used as a loading control (**p *<* 0.01, ***p *<* 0.001, one-way analysis of variance, NS: non-significant). *N* = 3 independent experiments with triplicate biological replicates for each line

ATC cell lines (8595C, HTH7, and FRO) and normal thyroid cells (Nthy-ori 3-1) were used for the comparison of Pard3 mRNA and protein expressions. When compared with normal cells, Fig. 7a shows that the expression of Pard3 mRNA was significantly lower than in ATC cells. Figure 7b shows that to determine if Pard3 knockdown regulates normal cells, Pard3 expression in FRO cells was silenced using three short hairpin RNAs (shRNAs). Supplementary Figure 1e–h shows that the cell growth, migration, and invasion of normal thyroid Nthy-ori 3-1 cells were increased when Pard3 was knocked down. Cell IHC analyses showed that the intensity of Pard3 staining in cells corresponding to Pard3 mRNA expression was also lower in ATC cells than normal cells (Supplementary Figure 2a). Moreover, knockdown with three shRNAs and Pard3 staining levels were also detected by IHC analyses in FRO cells, showing that knockdown of Pard3 increased FRO cell proliferation (Supplementary Figure 2b–d). In addition, overexpressed Pard3 mRNA and protein expression and Pard3 staining by IHC were also detected in 8505C cells, and overexpression of Pard3 inhibited cell proliferation (Supplementary Figure 2e–h). Pard3-shRNA3 had the highest knockdown efficiency, and therefore, was used for subsequent experiments. We confirmed Pard3 protein knockdown in FRO cells by western blotting (Fig. 7c). Pard3 knockdown significantly downregulated E-cadherin expression and upregulated vimentin expression (Fig. 7c). We also detected the other EMT-related proteins, N-cadherin, Snail, Twist, and ZEB1 (Supplementary Figure 2i), to show that Pard3 knockdown promoted the EMT. In addition, Pard3 knockdown activated Tiam1-Rac1 signaling in ATC cells (Fig. 7d, e). The experiments were also replicated with Pard3-shRNA2, and the results were similar, but with a reduced significance (Supplementary Figure 2j–m).

We then determined how the overexpression and knockdown of Pard3 might affect the TGF-β1-induced EMT in 8505C and FRO cells. Vimentin expression was increased, E-cadherin was reduced, and Tiam1 and Rac1 expressions were increased when ATC cells were treated with TGF-β1. The results indicated that this treatment promoted Tiam1-Rac1 signaling and the EMT (Fig. 7f–k). In addition, the EMT-related markers N-cadherin, Snail, Twist, and ZEB1 were upregulated when cells were treated with TGF-β1 (Supplementary Figure 3a, c). These effects were inhibited by Pard3 overexpression and enhanced by Pard3 knockdown (Pard3-shRNA3) (Fig. 7f–k). Supplementary Figure 3b, d shows that immunofluorescence confirmed the effects of TGF-β1 and Pard3 on the EMT marker N-cadherin and E-cadherin protein expression. Supplementary Figure 4 shows the use of Pard3-shRNA2 to determine the effects on TGF-β1-induced EMT in FRO cells, whose results were similar to those of Pard-shRNA3, but with a reduced significance.

### MiR-483 activated EMT and Tiam1/Rac1 signaling by inhibiting Pard3 expression

The influence of miR-483 on TGF-β1-mediated EMT and Tiam1-Rac1 signaling via Pard3 was then determined. We introduced miR-483 inhibitors and mimics into TGF-β1-treated ATC cells, in which Pard3 expression was upregulated or downregulated. As shown previously, Pard3 overexpression reversed the TGF-β1-mediated effects on E-cadherin, and vimentin (Fig. 7l, o), and on N-cadherin, Snail, Twist, and ZEB1 expression (Supplementary Figure 5a), and inhibiting miR-483 reversed these effects, while miR-483 mimics enhanced these effects in ATC cells (Fig. 7l, o). Supplementary Figure 5b shows the influence of miR-483 on Pard3-TGFβ1-mediated E-cadherin and N-cadherin protein expressions, using immunofluorescence. Figure 7m, n, p, q show that downregulation of TGF-β1-induced Tiam1-Rac1 signaling by Pard3 in ATC cells, which was prevented by miR-483 inhibitors, and was increased by treatment with miR-483 mimics. The expression of Pard3 was relatively higher in FRO cells, so we determined the knockdown by Pard3-shNRA2/3 in TGF-β1-treated FRO cells, and then transfected them with either miR-483 mimics or inhibitor. Knockdown of Pard3 increased the TGF-β1-induced EMT and Tiam1/Rac1 signaling while rescuing the effects of miR-483 inhibitor on FRO cells (Supplementary Figures 6 and 7). Overall, the results showed that inhibiting Pard3 by miR-483 treatment activated Tiam1/Rac1 signaling and the EMT.

### MiR-483 increases ATC cell migration and invasion by inhibiting Pard3

**Fig. 8:**
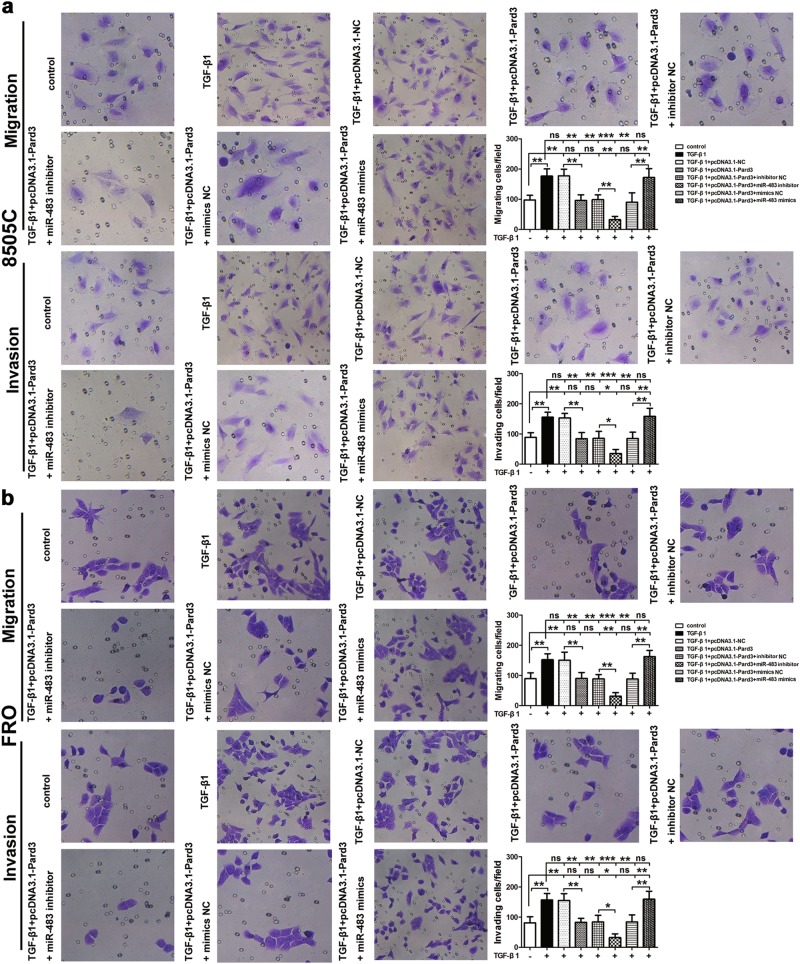
MiR-483 promotes TGF-β1-induced cell migration and invasion by downregulating Pard3. ATC cells stably transfected with pcDNA3.1-NC or pcDNA3.1-Pard3 were transfected with miR-483 inhibitor/miR-483 inhibitor NC, or miR-483 mimics/miR-483 mimics NC, and subsequently treated with TGF-β1 (10 ng/mL) for 48 h. Untransfected cells with or without TGF-β1 treatment were also included. The 8505C (**a**) and FRO (**b**) cell migration and invasion were measured by transwell assays (*p < 0.05, **p < 0.01, ***p < 0.001, one-way analysis of variance; ns: non-significant). *N* = 3 independent experiments with triplicate biological replicates for each line

Transwell assays were then used to determine the effect of miR-483-mediated Pard3 regulation on TGF-β1-induced migration and invasion of FRO and 8505C cells. Figure 8 shows that treatment with TGF-β1 resulted in increased migration and invasion of ATC cells, while these effects were inhibited when Pard3 was overexpressed. Supplementary Figure 8 shows that although Pard3 knockdown increased the migration and invasion of FRO cells, it also rescued the effects of the miR-483 inhibitor on migration and invasion of cells. The inhibition of miR-483 resulted in the enhancement of Pard3-mediated inhibition of cell migration and invasion. However, TGF-β1-induced cell migration and invasion of ATC cells were increased by miR-483 treatment. Taken together, the results suggested that inhibition of Pard3 resulted in the promotion of ATC cell migration and invasion by miR-483.

### Pard3 inhibits the effects of miR-483 on cell EMT and Tiam1/Rac1 signaling and cell migration and invasion independent of TGF-β1 signaling

We then investigated whether miR-483 overexpression increased EMT expression by downregulating Pard3 independent of TGF-β1 signaling. The 8505C cells overexpressing Pard3 were able to inhibit the effects of miR-483 mimics on EMT and Tiam1/Rac1 signaling and cell migration and invasion in the presence of a TGF-β1 inhibitor (Supplementary Figure 9), while Pard3-shRNA3 rescued the effect of miR-483 inhibitor on EMT and Tiam1/Rac1 signaling and cell migration and invasion in the presence of a TGF-β1 inhibitor (Supplementary Figure 10). These results were confirmed by assessment of E-cadherin and N-cadherin expression in 8505C and FRO cells by immunofluorescence (Supplementary Figures 9d and 10d).

## Discussion

The effects of miR-483 and Pard3 on the TGF-β1-induced progression of thyroid cancer were characterized in the present study. Activated TGF-β1 receptors recruit Pard3 to control cell polarity and promote cell migration in developing orofacial tissue [24]. Furthermore, migration and tumorigenesis in various cancer cell types are promoted by the expression of miRNAs, which is regulated by treatment with TGF-β1 [11–14]. In the present study, the expression of miR-483 was increased, while the expression of Pard3 was reduced in ATC cells and tissues. The results showed that after binding of Pard3 and miR-483, the miR-483 downregulated Pard3 in thyroid carcinomas. In addition, the miR-483 promotion of TGF-β1-induced thyroid cancer cell proliferation, migration, and invasion, both in vitro and in vivo, was possibly mediated by the downregulation of Pard3. Although the exact mechanism of action will require future studies, increased levels of TGF-β1-induced Smad2 and Smad3 activity, resulting in increased levels of miR-483.

Thyroid carcinoma tissues contain Tiam1, which is a Rac1-specific GEF that is overexpressed in thyroid carcinomas. Pard3 interacts with Tiam1 to increase Rac1 activation. The migration and invasion of thyroid cancer cells may be influenced by miR-483, in a mechanism involving regulation of Pard3 expression and Tiam/Rac1 signaling. Different aspects of tumor development are influenced by overexpression of Tiam1 in many types of tumors. In both breast cancer [25] and colorectal carcinoma cells [26], there was a positive correlation between the migration and metastatic potential of tumor cells and the expression of Tiam1. However, the expression of Tiam1 was correlated negatively with the invasion of renal cell carcinomas [27], and downregulation of Tiam1 was present during breast cancer progression [28]. Liu et al. [29] found that cell migration and invasion, as well as metastasis of carcinomas, was increased after Tiam1 treatment. How Tiam1 is involved in tumor formation and metastasis, as well as the parameters that affect its expression, are unclear. In the current study, we showed that Tiam1/Rac1 signaling was regulated by Pard3 in thyroid carcinomas.

TGF-β1 regulates a wide variety of biological activities. Numerous studies have proved that TGF-β1 induces migration and invasion in cancers, such as hepatocellular carcinoma [30], colorectal cancer [31], salivary adenoid cystic carcinoma [32], osteosarcoma [33], and thyroid cancer [34]. TGF-β1 can induce EMT [35], which promotes invasion and migration in cancer. During EMT, epithelial cells acquire a migratory behavior by losing their epithelial characteristics, such as cell polarity and specialized cell–cell contacts [35]. The key event in EMT is E-cadherin to N-cadherin conversion, which renders single cells more motile and invasive [36]. In the present study, the invasive and migratory phenotype of the 8505C and FRO cell lines obtained by Transwell assay were corroborated by the use of the EMT markers N-cadherin, Snail, Twist, and ZEB1. Indeed, miR-483 promoted TGF-β1-induced EMT and cell migration, but could influence Pard3 independently. Silencing miR-483 reversed the effects of TGF-β1 on EMT progression and cell migration. When Pard3 was silenced, this induced miR-483-mediated cell proliferation, migration, and invasion in vitro, which might have occurred by Tiam/Rac1 signaling. Loss of Pard3 has been shown to promote tumorigenesis in different human cancers via Tiam1/Rac1-dependent mechanisms [18–22]. We have shown in the current study that when Pard3 was downregulated, this induced the expression of EMT ATC cells that was independent of treatment by TGF-β1. In contrast, in breast cancer, the loss of Pard3 promoted metastasis and decreased cell–cell cohesion, which occurred in the absence of EMT induction [19]. Furthermore, Zhou et al. [37] reported that downregulation of Pard3 increased lung adenocarcinoma cell EMT and invasion. We characterized the downregulation of Pard3 in ATC cells, a process that has a poor prognosis. The prognosis for ATC is very poor, with approximately 6 months survival after the diagnosis [38]. Neither chemotherapy nor radiation therapy are effective at prolonging the survival of ATC patient [39]. We have reported in a preliminary study that overexpression of miR-483 resulted in an increase in cell migration and invasion, and also resulted in the induction of EMT in lung cancer [10]. During this process, it was also found that miR-483 was upregulated and Pard3 was downregulated in ATC cells. The use of a luciferase assay showed the direct binding of miR-483 and Pard3, resulting in decreased expression of Pard3. Furthermore, the knockdown of miR-483 was involved in the suppression of Pard3-mediated inhibition of ATC cell invasion. Pard3 inhibits Tiam1/Rac1-induced actin polymerization, thereby stabilizing cell–cell contacts and suppressing cell growth and migration. The increased cell migration and invasion may have resulted from the downregulation of Pard3 by miR-483 in thyroid tumor tissues following overexpression of miR-483. We also showed in a recent study [29] that Tiam1 overexpression resulted in the induction of EMT, together with an increase in cell migration and invasion, which was dependent on the activation of Rac1. Therefore, we predicted that knockdown of Pard3 may cause the induction of EMT in ATC cells via the Tiam-Rac1 signaling pathway in ATC cells. However, more functional studies are necessary to identify the exact mechanism of actions.

Tiam1 expression is regulated by a number of factors including c-myc [40], Raf/MAPK activation [41], and 14-3-3 proteins [42]. In addition, miRNAs regulate Tiam1-mediated migration and invasion of hepatomas [43], colon carcinomas [44], nasopharyngeal carcinomas [45], breast cancer [46], and osteosarcomas [47]. Our findings showed that miR-483 upregulated Tiam1/Rac1 signaling in thyroid carcinomas, possibly by downregulating Pard3. Tiam1 upregulation promotes intestinal tumor formation and progression [26] via a feedback loop; aberrant Wnt activation induces Tiam1 transcription, thereby activating Rac1 [26]. Enhanced Tiam1/Rac1 signaling may increase the transcription of Wnt target genes to promote tumor initiation and progression.

Overall, the results of the present study have shown that Pard3 inhibited TGF-β1-induced cell EMT and invasion, concomitant with miR-483 downregulating Pard3 in thyroid carcinoma cells, to activate Tiam/Rac1 signaling and promoting EMT, as well as cell migration and invasion. This may promote tumorigenesis of thyroid carcinomas by destabilizing cell–cell contacts and promoting cell migration and invasion. Taken together, our results increased our basic knowledge of the mechanisms involved in thyroid cancer formation, which will hopefully assist in the development of novel agents to treat thyroid cancer.

## Materials and methods

### Patient tissues

Between 2010 and 2013, 80 pairs of tissues, representing thyroid cancer tissues and adjacent normal thyroid tissues were obtained after resection of thyroid carcinomas or thyroid nodules from patients at Shanghai Tenth People’s Hospital Affiliated with Shanghai Tongji University. Preoperative chemotherapy was not performed on any of the patients. The Research Ethics Committee of Shanghai Tenth People’s Hospital (Shanghai, China) approved the resection of all specimens. Tissue sections were inspected by three pathologists to confirm the diagnosis and to identify the tissue variants, which were comprised of 33 papillary carcinomas, 25 follicular carcinomas, 13 poorly differentiated carcinomas, and nine anaplastic carcinomas (Table 1).The tissues were frozen in liquid nitrogen after surgical removal and stored at −80 °C until analyzed.

### Cell culturing and reagents

The American Type Culture Collection (Manassas, VA, USA) and the Shanghai Institute of Cell Biology (Shanghai, China) were the sources of all human ATC cell lines, including FRO, 8505C, HTH7, and the Nthy-ori 3-1 thyroid follicular cell line. RPMI-1640 medium (Gibco, Carlsbad, CA, USA) was used to culture the Nthy-ori 3-1 cells. Dulbecco’s modified Eagle’s medium (Gibco) was used to culture the ATC cells. The culture medium also contained 10% fetal bovine serum (FBS; Invitrogen-Gibco), 100 U/mL sodium salt of penicillin G, and 100 U/mL streptomycin sulfate (Invitrogen-Gibco), and all cells were grown at 37 °C in an atmosphere of 5% CO_2_. The Vendor GeM Mycoplasma Detection Kit (Minerva Biolabs, Berlin, Germany) was used to test all cell lines for mycoplasma contamination. R&D Systems (Minneapolis, MN, USA) was the source of the human recombinant TGF-β1, and Sigma-Aldrich (St. Louis, MO, USA) was the source of the TGF-β receptor kinase inhibitor, SB431542.

### The assay using quantitative real-time PCR

TRIzol reagent (Invitrogen) was used to isolate RNA according to the instructions of the manufacturer. The ABI PRISM 7500 Sequence Detection System (Applied Biosystems, Foster City, CA, USA), with a SYBR Premium Ex Taq II Kit (Takara, Dalian, China), was used for the quantitative real-time PCR (qRT-PCR). The Pard3 primers were the following: forward, 5′-CACACGCTGGTCAACAACAG-3′ and reverse, 5′-GGTGACTGGCACTCAGAGAC-3’. The control was glyceraldehyde 3-phosphate dehydrogenase (GAPDH). The TaqMan MicroRNA Assay Kit (Applied Biosystems) was used to analyze miR-483, and the expression of miR-483 was determined according to the previous studies [48]. The following primers were used for miR-483: RT primer, 5′-GTCGTATCCAGTGCAGGGTCCGAGGTATTCGCACTGGATACGACAAGACG-3’, forward primer, 5′-GGCTAGTTCACTCCTCTCCTCC-3′, reverse primer, 5′- GTGCAGGGTCCGAGGT-3′; and U6, RT primer, 5′-CGCTTCACGAATTTGCGTGTCAT-3′, forward primer, 5′-GCTTCGGCAGCACATATACTAAAAT-3′, reverse primer, 5′-CGCTTCACGAATTTGCGTGTCAT-3′. The 2^−ΔΔCT^ method [49] was used to measure the relative expression.

### Immunohistochemistry

Tissue samples were prepared for immunohistochemical staining according to a previous study [50] Anti-Pard3 (ab64646, 1:200) antibody was used to incubate tissue sections overnight at 4 °C. Previously described methods [51, 52] were used for the analyses of the intensity and distribution of Pard3 immunostaining. Staining intensity was scored as 0 (no staining), 1 (weak staining), 2 (moderate staining), and 3 (strong staining), and the percentage of positive tumor cells (0–100%) was used to measure the distribution of Pard3 expression was calculated by multiplying the intensity and distribution. “Low” (Pard3 low) and “high” (Pard3 high) were used to characterize Pard3 expression, using the cutoff point from the X-tile software program [53].

### The construction and transfection of plasmids

Three small, interfering RNAs were used to knockdown Pard3. Their constructions were as follows: *Pard3*-shRNA1, 5′-CGTGGAGTCTTACATAAAT-3′; shRNA2, 5′-CGACACTACTACTCAATTA-3′; shRNA3, 5′-CCCGTTCTGTTTGGTTCTA-3′; and the scrambled negative shRNA, 5′-GACCTGTACGCCAACACAGTG-3′, was used as a control. GeneChem (Shanghai, China) was used for the chemical synthesis of the shRNA, followed by their subcloning into the pSilencer 4.1 vector (Invitrogen, NY, USA). Stable cell lines were selected using 3 μg/mL puromycin (Gibco, Grand Island, NY, USA). To overexpress *Pard3*, a full-length cDNA was amplified by using the following primers: forward, 5′-GCGGGTACCATGAAAGTGACCGTGTGCTTC-3′; and reverse, 5′-GGCCTCGAGTCAGGAATAGAAGGGCCTCCC-3′. The *Kpn*I and *Xho*I sites (TaKaRa, Dalian, China) were used to insert the *Pard3* cDNA product into the pcDNA3.1(+) vector (Invitrogen). We used G418 to select the stable colonies.

### Transfection with miR-483 mimics and inhibitors

Two scrambled miRNAs were used as negative controls (NCs; mimics NC for miR-483 mimics and inhibitor NC for the miR-483 inhibitor, respectively), which were purchased from GeneChem (Shanghai, China) and used for the overexpression and knockdown of miR-483, miR-483 mimics, and the miR-483 inhibitor. The 100 nM miR-483 mimics and inhibitors were transfected into FRO and 8505C cells using Lipofectamine RNAiMAX reagent (Invitrogen).

### Lentivirus construction

As previously described [54], the overexpression of miR-483, miR-483 inhibitor, or corresponding control oligonucleotides were cloned into pLVX vectors (Clontech, Mountain View, CA, USA) at the *Xho*I and *Eco*RI sites downstream of the green fluorescent protein (GFP) site. Human embryonic kidney 293T (HEK293T) cells were transfected with an miR-483-overexpressing lentivirus (LV-miR-483), a miR-483 inhibitor lentivirus (LV-anti-miR-483), or a control lentivirus, together with a Lenti-X HTX packaging mix (Clontech) using Lipofectamine 2000. After 48 h, the viruses were collected. HEK293T cells (5 × 10^5^) were seeded into 12-well plates for virus titer determinations, and then infected with a series of lentivirus dilutions. The number of GFP-positive cells (10 fields/well) were counted after infection for 48 h, using a fluorescence microscope to detect the viruses. We added puromycin (2 μg/mL) to select stably transfected cells.

### The assay for cell proliferation

The Cell Counting Kit-8 (CCK-8, Dojindo, Kumamoto, Japan) was used to quantitate cell proliferation, using the manufacturer’s instructions as previously described [22]. In brief, ATC cells (2000 cells/well) were seeded into 96-well plates, followed by overnight culturing. Then, 10 µL of CCK-8 reagent was added to quantitate cell proliferation at 1–5 days by measuring the *A*_450 nm_ using an Epoch Microplate spectrophotometer (BioTek, Winooski, VT, USA).

### The assays for cell migration and invasion

We used 24-well inserts (BD Bioscience, Bedford, MA, USA) to plate ATC cells. The plates were either uncoated for migration assays or coated with Matrigel for the invasion assays. The upper chamber contained 2 × 10^4^ cells/well in 100 μL serum-free medium, and the lower chamber contained 600 μL in 10% FBS serum medium as a chemoattractant. The cells were incubated for 19 h, and a cotton swab was then used in the upper chamber to remove non-migrating cells. Cells that migrated to the lower chamber were quantitated using five random fields by visualization with a microscope at ×200 magnification (Nikon, Tokyo, Japan).

### Western blot analyses

Radioimmunoprecipitation assay buffer containing protease inhibitors was used to lyse ATC cells. Sodium dodecyl sulfate-polyacrylamide gel electrophoresis was used to resolve total protein extracts, followed by protein transfer to nitrocellulose membranes. The membranes were blocked with 5% non-fat milk for 1 h, and were then incubated overnight at 4 °C with primary antibodies. The primary antibodies were the following: anti-Pard3 (ab64646, 1:500), anti-Rac1 (ab97732, 1:500), anti-Tiam1 (ab211518,1:1000), anti-E-cadherin (ab40772, 1:5000), anti-vimentin (ab92547, 1:1000), anti-N-cadherin (ab18203, 1:1000), anti-Snail (ab82846, 1:500), anti-Twist (ab211518, 1:1000), anti-ZEB1 (ab124512, 1:500), anti-phospho-Smad2 (ab53100, 1:1000), anti-Smad2 (ab40855, 1:1000), anti-phospho-Smad3 (ab52903, 1:1000), and anti-Smad3 (ab84177, 1:1000) (all from Abcam, San Francisco, CA, USA). The membranes were washed, and then treated for 1 h at 37 °C with goat anti-rabbit IgG conjugated with horseradish peroxidase (HRP). Protein bands were visualized by using a chemiluminescence HRP substrate, and GAPDH was used as an internal control.

### The activity assays for Rac1-GTPase and Tiam1

The activity assays for Rac1-GTPase and Tiam1 were performed as previously described [55]. The p21-activated protein kinase agarose beads were used to pull down the active Rac1 by using a GTPase Activation Assay Kit (STA-401-1; Cell Biolabs, San Diego, CA, USA). Rac1 G15A agarose beads were used to pull down the active Tiam1, followed by use of an assay kit for active Rac-GEF (STA-422; Cell Biolabs).

### Immunofluorescence staining

Overexpressed Pard3 (pcDNA3.1-Pard3) or knockdown Pard3 (Pard3-shRNA2 or Pard3-shRNA3) was used for transfection, with co-transfection using miR-438 mimics or miR-483 inhibitors. TGF-β1 (10 ng/mL) was used for transfection and treated for 48 h. Formalin (4%) was then used to fix the cells, followed by a phosphate-buffered saline wash, and permeabilization using 0.1% Triton X-100. The cells were then treated with primary antibodies against E-cadherin (ab40772, 1:100) and N-cadherin (ab98952, 1:100) overnight at 4 °C, and then treated with a secondary goat anti-rabbit IgG H&L (FITC) (ab6717) or goat anti-mouse IgG H&L (Cy3) (ab97035) for 1 h at 37 °C. The cells were then stained with DAPI (4′,6-diamidino-2-phenylindole) and visualized with a confocal microscope.

### Dual-luciferase reporter assay

The following primers: wt, 3′-UTR of Pard3 (Pard3-wt), forward, 5′-CGCCTCGAGTTCCTGACACGTGGGTTGAG-3′ and reverse, 5′-CGGGCGGCCGCCTGTCCCATTCTGTGCCCTT-3′; mutant 3′-UTR of Pard3 (Pard3-mut), forward, 5′-CGCCTCGAGTGGGAGACACCTCAGGCTC-3′ and reverse, 5′-CGGGCGGCCGCTGTCCCATTCTGTGCCCTTC-3′ were used to amplify the 3′-UTR cDNA fragments of Pard3 containing the putative wt or mutant miR-483 binding sites. The *Xho*I and *Not*I sites of the psiCHECK-2 vector (Promega, Madison, WI, USA) were used to subclone the amplified cDNA fragments. The 293T cells were then co-transfected with the luciferase reporter system and miR-483 mimics/miR-NC by incubating them for 48 h. Then, the activity of luciferase was determined using the manufacturer’s instructions for the Dual-Luciferase Reporter Assay System (Promega). The control was the Renilla luciferase activity.

### The tumorigenicity assay of the tumor xenograft model

The xenograft tumor model was used as previously described [20]. Nude male BALB/cA-nu mice (6 weeks of age) were provided by the Shanghai SLAC Laboratory Animal Co., Ltd. (Shanghai, China). The animals were randomly assigned to three groups (*n* = 6/group). The Ethics Committee of Shanghai Tenth People’s Hospital of China (Shanghai, China), the Principles of Laboratory Animal Care (National Society for Medical Research), and the National Institutes of Health guidelines were used to design all animal studies. LV-miR-483 or LV-anti-miR-483 were used to infect 2 × 10^6^ 8505C or FRO cells. Tumor formation was initiated by subcutaneous injection of infected ATC cells into the flanks of nude mice. The formula, volume = length × width^2^/2, was used to calculate the tumor volume from measurements taken every 3 days. The tumor grafts were excised, weighed, and harvested after euthanizing the mice 27 days after injection.

## Statistical analysis

Results are expressed as the mean ± SD from the results of three independent experiments performed in triplicate. The Student’s *t* test was used to determine significant differences between two groups, and one-way analysis of variance was used for multiple groups followed by Dunnett’s multiple comparison test or Bonferroni’s multiple comparison test. A value of *p* < 0.05 was considered significant. The sample size was adjusted to achieve maximum statistical power. Pearson’s *χ*^2^ test was used to identify Pard3 expression that correlated with clinicopathological parameters. The Kaplan–Meier method was used to generate survival curves and the log-rank test was used for statistical analyses. As previously reported [56–58], 95% confidence was considered significant. SPSS statistical software for Windows, version 17.0 (SPSS, Chicago, IL, USA) was used for all statistical analyses. The analyses included data from all animal studies, and the investigators were blinded to the identity of the animals.

## Electronic supplementary material

supplementary figure 1

supplementary figure 2

supplementary figure 3

supplementary figure 4

supplementary figure 5

supplementary figure 6

supplementary figure 7

supplementary figure 8

supplementary figure 9

supplementary figure 10
