# Supplementary material for: MicroRNA 483-3p targets Pard3 to potentiate TGF-β1-induced cell migration, invasion, and epithelial–mesenchymal transition in anaplastic thyroid cancer cells
Source: Oncogene. 2018 Aug 31;38(5):699–715. doi: 10.1038/s41388-018-0447-1 (PMC6756112; doi:10.1038/s41388-018-0447-1)
Supplement: Supplementary file 1 — supplementary figure 1 [file 41388_2018_447_MOESM1_ESM.pdf]

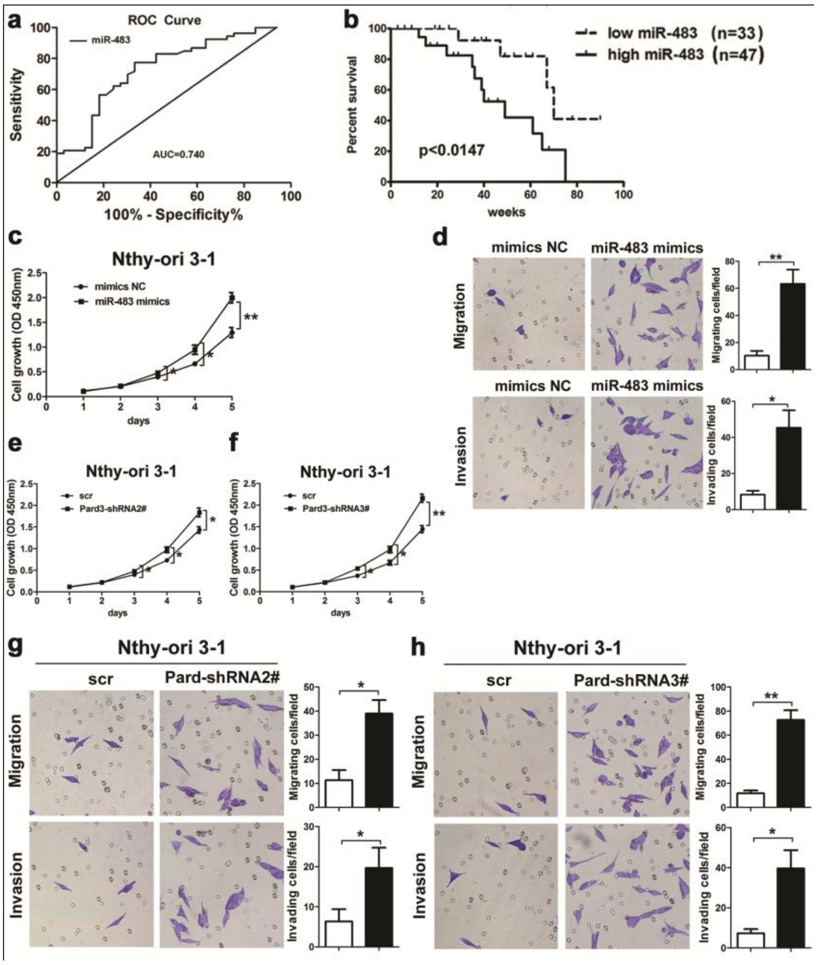

Supplementary Figure 1. High MiR-483 decreases lower survival in patients and increases normal cell growth, migration and invasion and Pard3 knockdown increases normal thyroid cell growth, migration and invasion. **(a)** Receiver operating characteristics (ROC) curve analyzed miR-483 in thyroid cancer tissues. MiR-483 yielded an AUC (the areas under the ROC curve) of 0.740 (95 % CI 0.631 –0.850; p = 0.002). (**b**) Kaplan–Meier analyzed the relationship of miR-483 expression and survival of thyroid cancer patients. (**p* < 0.0147, Log-rank test). **N**thy-ori 3-1 cells were transfected with mimics NC and miR-483 mimics, or scr and Pard-shRNA2#/3#, respectively. (**c, e, f**) Cell growth was determined by the CCK-8 assay (**p* <0.05, ***p* <0.01, one-way ANOVA). (**d, g, h**) Cell migration and invasion were assessed by Transwell assays (**p* <0.05, ***p* <0.01, Student's *t*-test). N = 3 independent experiments with triplicate biological replicates for each line.
