# Supplementary material for: MicroRNA 483-3p targets Pard3 to potentiate TGF-β1-induced cell migration, invasion, and epithelial–mesenchymal transition in anaplastic thyroid cancer cells
Source: Oncogene. 2018 Aug 31;38(5):699–715. doi: 10.1038/s41388-018-0447-1 (PMC6756112; doi:10.1038/s41388-018-0447-1)
Supplement: Supplementary file 2 — supplementary figure 2 [file 41388_2018_447_MOESM2_ESM.pdf]

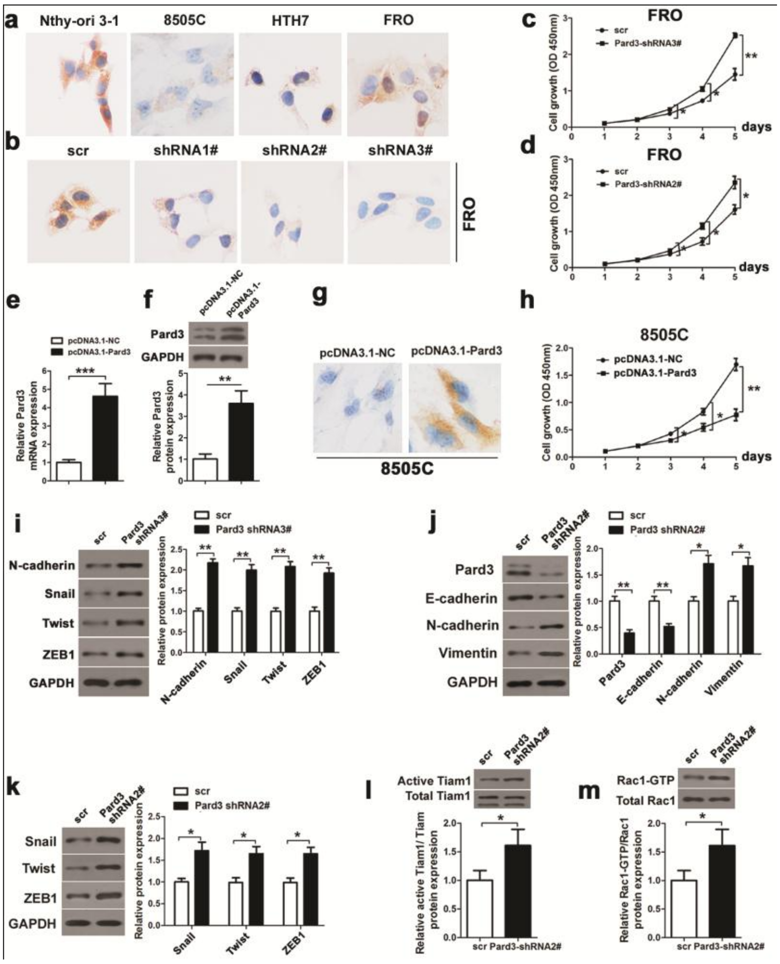

Supplementary Figure 2. Knockdown of Pard3 activates EMT expression. (**a**) The expression of Pard3 by IHC analysis in cell lines normal thy-ori 3-1, 8505C, HTH7, and FRO cells. (**b**) The expression of Knockdown of Pard3 (Pard3 shRNA1#–3#) in FRO cells was analyzed by IHC. (**c, d**) FRO cells were transfected with Pard3-shRNA2# or shRNA-3# or scr. Cell growth was determined by the CCK-8 assay (**p* <0.05, ***p* < 0.01, one-way ANOVA). (**e, f**) Overexpression of Pard3 (pcDNA3.1-Pard3) was detected by qRT-PCR and western blot analysis in 8505C cells (***p* < 0.01, *** *p* < 0.001, Student's *t*-test). (**g**) The expression of overexpression of Pard3 (pcDNA3.1-Pard3) in 8505C cells was analyzed by IHC. (**h**) 8505C cells were transfected with pcDNA3.1-Pard3 or pcDNA3.1-NC. Cell growth was determined by the CCK-8 assay (**p* <0.05, ***p* < 0.01, one-way ANOVA). **(i)** FRO cells were stably transfected with Pard3-shRNA3# or scr. N-cadherin, Snail, Twist and ZEB1 expression were detected by western blot. GAPDH was used as a loading control (***p* < 0.01, Student's *t*-test). (**j-m**) FRO cells were stably transfected with Pard3-shRNA2# or scr. E-cadherin, Vimentin, N-cadherin, Snail, Twist and ZEB1, active Tiam1 and Rac1 expression were detected by western blot. GAPDH was used as a loading control (**p* < 0.05, ***p* < 0.01, Student's *t*-test). N = 3 independent experiments with triplicate biological replicates for each line.
