# Supplementary material for: MicroRNA 483-3p targets Pard3 to potentiate TGF-β1-induced cell migration, invasion, and epithelial–mesenchymal transition in anaplastic thyroid cancer cells
Source: Oncogene. 2018 Aug 31;38(5):699–715. doi: 10.1038/s41388-018-0447-1 (PMC6756112; doi:10.1038/s41388-018-0447-1)
Supplement: Supplementary file 3 — supplementary figure 3 [file 41388_2018_447_MOESM3_ESM.pdf]

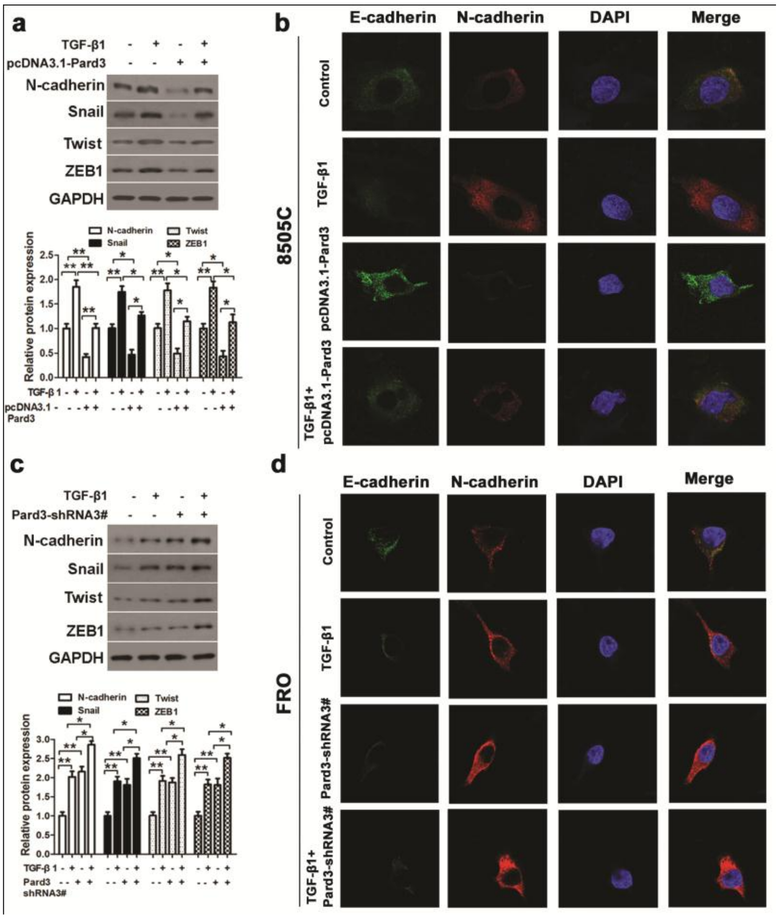

Supplementary Figure 3. Pard3 regulates TGF-β1 induced ATC EMT. 8505C cells were stably transfected with pcDNA3.1-Pard3 and subsequently treated with TGF-β1 (10 ng/ml) for 48 h. Untransfected cells with or without TGF-β1 treatment were also included. (**a**) N-cadherin, Snail, Twist and ZEB1 expression were detected by western blotting. GAPDH was used as a loading control (*p < 0.05, **p < 0.01, one-way ANOVA). (**b**) E-cadherin and N-cadherin expression in 8505C was detected by immunofluorescence. FRO cells were transfected with Pard3 shRNA3#, and subsequently treated with TGF-β1 (10 ng/ml) for 48 h. Untransfected cells with or without TGF-β1 treatment were also included. (**c**) N-cadherin, Snail, Twist and ZEB1, expression were detected by western blotting. GAPDH was used as a loading control (*p < 0.05, **p < 0.01, one-way ANOVA). (**d**) E-cadherin and N-cadherin expression in FRO was detected by immunofluorescence. N = 3 independent experiments with triplicate biological replicates for each line.
