# Supplementary material for: MicroRNA 483-3p targets Pard3 to potentiate TGF-β1-induced cell migration, invasion, and epithelial–mesenchymal transition in anaplastic thyroid cancer cells
Source: Oncogene. 2018 Aug 31;38(5):699–715. doi: 10.1038/s41388-018-0447-1 (PMC6756112; doi:10.1038/s41388-018-0447-1)
Supplement: Supplementary file 4 — supplementary figure 4 [file 41388_2018_447_MOESM4_ESM.pdf]

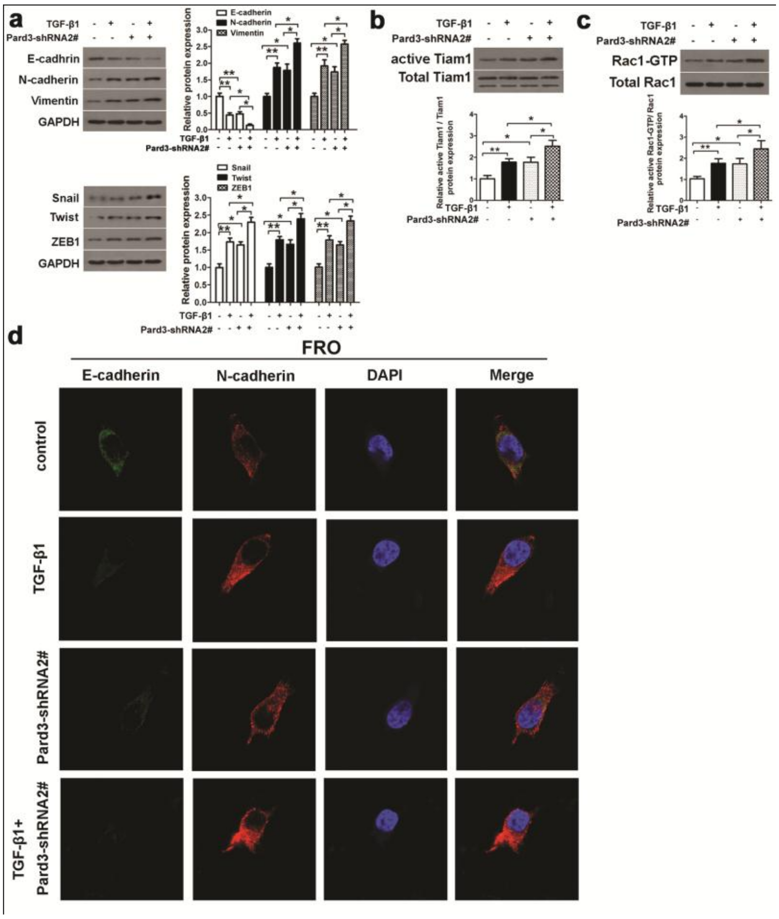

Supplementary Figure 4. Knockdown by Pard3-shRNA2# increases TGF-β1 induced ATC EMT and Tiam1/Rac1 signaling. FRO cells were transfected with Pard3 shRNA2#, and subsequently treated with TGF-β1 (10 ng/ml) for 48 h. Untransfected cells with or without TGF-β1 treatment were also included. (**a-c**) E-cadherin, N-cadherin, Vimentin, Snail, Twist and ZEB1, active Tiam1 and Rac1 expression were detected by western blotting. GAPDH was used as a loading control (*p < 0.05, **p < 0.01, one-way ANOVA). (**d**) E-cadherin and N-cadherin expression in FRO was detected by immunofluorescence. N = 3 independent experiments with triplicate biological replicates for each line.
