# Supplementary material for: MicroRNA 483-3p targets Pard3 to potentiate TGF-β1-induced cell migration, invasion, and epithelial–mesenchymal transition in anaplastic thyroid cancer cells
Source: Oncogene. 2018 Aug 31;38(5):699–715. doi: 10.1038/s41388-018-0447-1 (PMC6756112; doi:10.1038/s41388-018-0447-1)
Supplement: Supplementary file 5 — supplementary figure 5 [file 41388_2018_447_MOESM5_ESM.pdf]

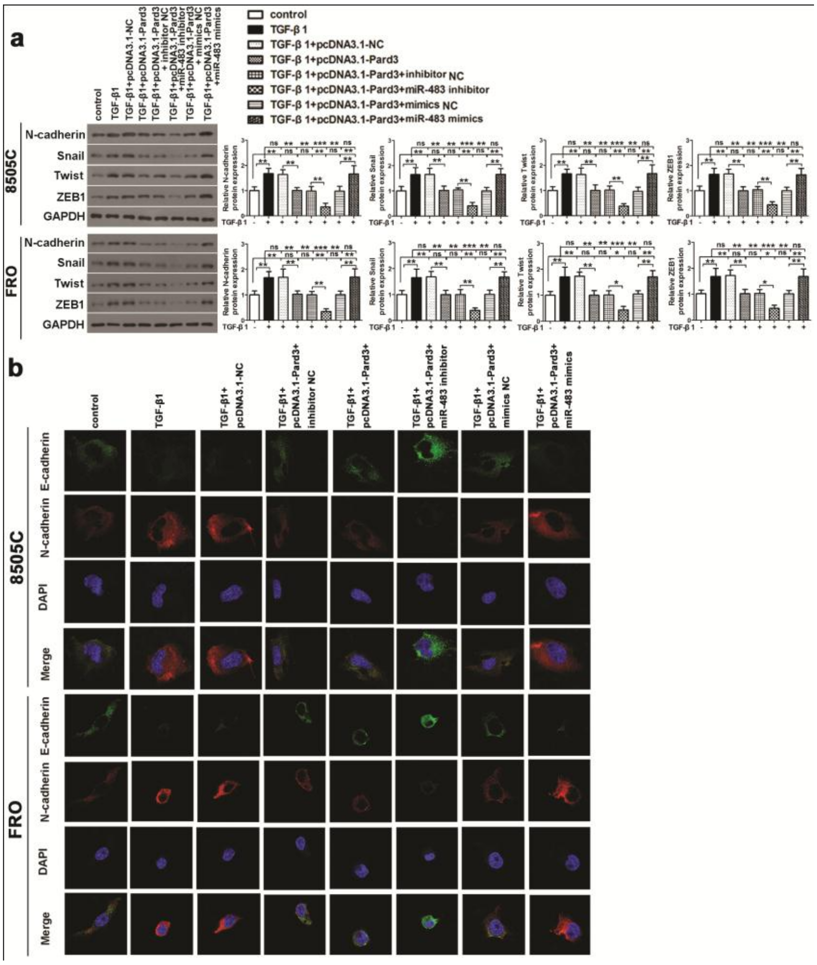

Supplementary Figure 5. MiR-483 promotes TGF-β1-induced EMT by downregulating Pard3 in ATC cells. 8505C and FRO cells stably transfected with pcDNA3.1-NC or pcDNA3.1-Pard3 were transfected with miR-483 inhibitor/ miR-483 inhibitor NC or miR-483 mimics/ miR-483 mimics NC and subsequently treated with TGF-β1 (10 ng/ml) for 48 h. Untransfected cells with or without TGF-β1 treatment were also included. (**a**) N-cadherin, Snail, Twist and ZEB1 expression were detected by western blotting. GAPDH was used as a loading control (**$p < 0.01$, ***$p < 0.001$, one-way ANOVA, ns= non-significant). (**b**) E-cadherin and N-cadherin expression in 8505C and FRO cells were detected by immunofluorescence. N = 3 independent experiments with triplicate biological replicates for each line.
