# Supplementary material for: MicroRNA 483-3p targets Pard3 to potentiate TGF-β1-induced cell migration, invasion, and epithelial–mesenchymal transition in anaplastic thyroid cancer cells
Source: Oncogene. 2018 Aug 31;38(5):699–715. doi: 10.1038/s41388-018-0447-1 (PMC6756112; doi:10.1038/s41388-018-0447-1)
Supplement: Supplementary file 8 — supplementary figure 8 [file 41388_2018_447_MOESM8_ESM.pdf]

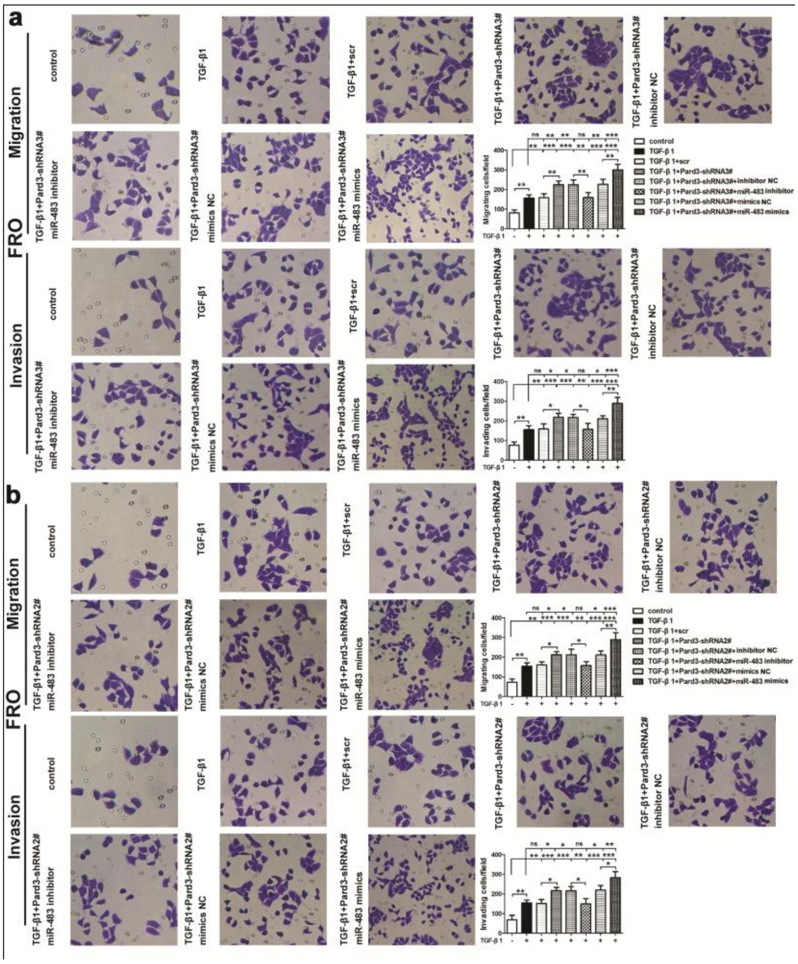

Supplementary Figure 8. Pard3 knockdown increases TGF-β1 induced FRO cells migration and invasion. FRO cells were transfected stably with Pard3-shRNA3# and 2#, miR-483 inhibitor/miR-483 inhibitor NC or miR-483 mimics/miR-483 mimics NC and subsequently treated with TGF-β1 (10 ng/ml) for 48 h. Untransfected cells with or without TGF-β1 treatment were also included. (**a, b**) FRO cell migration and invasion were measured by transwell assays (*$p < 0.05$, **$p < 0.01$, ***$p < 0.001$, one-way ANOVA, ns=non-significant). N = 3 independent experiments with triplicate biological replicates for each line.
