# Supplementary material for: MicroRNA 483-3p targets Pard3 to potentiate TGF-β1-induced cell migration, invasion, and epithelial–mesenchymal transition in anaplastic thyroid cancer cells
Source: Oncogene. 2018 Aug 31;38(5):699–715. doi: 10.1038/s41388-018-0447-1 (PMC6756112; doi:10.1038/s41388-018-0447-1)
Supplement: Supplementary file 9 — supplementary figure 9 [file 41388_2018_447_MOESM9_ESM.pdf]

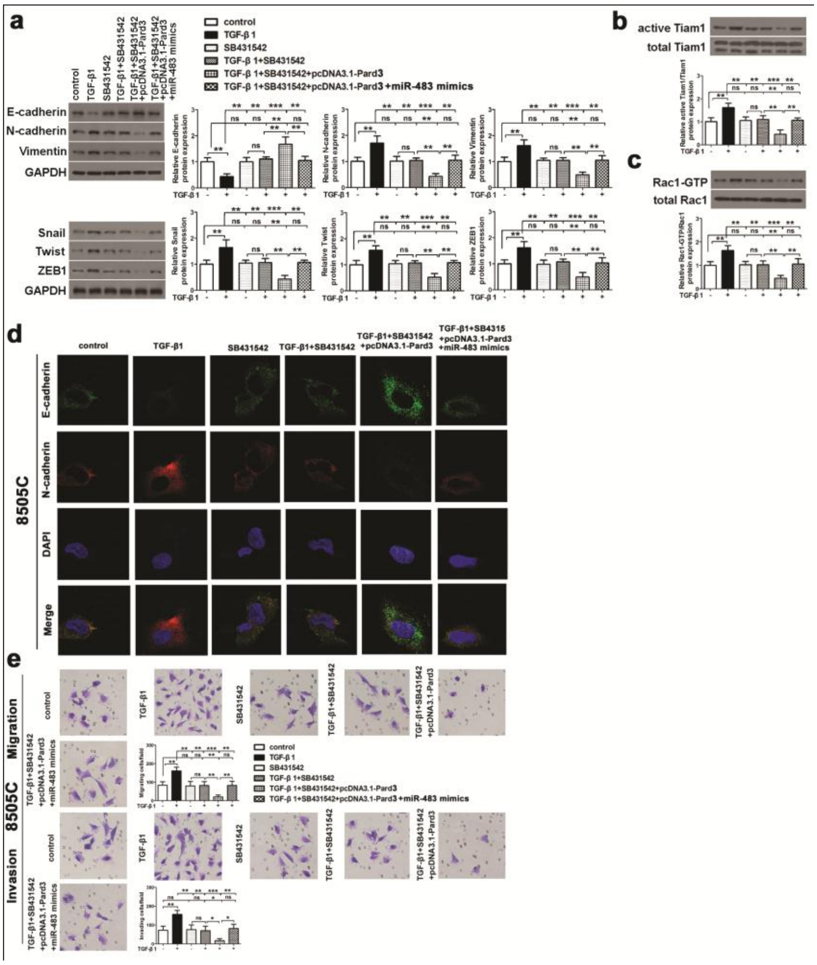

Supplementary Figure 9. overexpression miR-483 increases cell EMT and migration and invasion expression by downregulating Pard3 independent TGF-β1 signaling. 8505C Cells were treated with TGF-β1, TGF-β1 inhibitor (SB431542) or TGF-β1+SB431542, TGF-β1+SB431542+ pcDNA3.1-Pard3, or TGF-β1+ SB431542+ pcDNA3.1-Pard3+miR-483 mimics for 48 h. Untransfected cells were set as a control. (**a-c**) E-cadherin, N-cadherin, Vimentin, Snail, Twist and ZEB1, active Tiam1 and Rac1 expression were detected by western blotting. GAPDH was used as a loading control (***p* < 0.01, ****p* < 0.001, one-way ANOVA, ns= non-significant). (**d**) E-cadherin and N-cadherin expression in 8505C cells was detected by immunofluorescence. (**e**) 8505C cell migration and invasion were measured by transwell assays (**p* < 0.05, ***p* < 0.01, ****p* < 0.001, one-way ANOVA, ns=non-significant). N = 3 independent experiments with triplicate biological replicates for each line.
